# GSN synergies with actin-related transfer molecular chain to promote invasion and metastasis of HCC

**DOI:** 10.1007/s12094-022-02961-1

**Published:** 2022-10-03

**Authors:** Yi Zhou, Min He

**Affiliations:** 1 Life Sciences Institute, Guangxi Medical University, Nanning, 530021 China; 2 Key Laboratory of High-Incidence-Tumor Prevention & Treatment (Guangxi Medical University), Ministry of Education, Nanning, 530021 China; 3 Laboratory Animal Center, Guangxi Medical University, Nanning, 530021 China

**Keywords:** Gelsolin (GSN), HCC metastasis, Actin, Therapeutic target

## Abstract

**Background:**

Previous studies have shown that the ability of tumor cells to move and migrate is related to the molecular chain pathway mediated by actin. This study focused on the molecular mechanism of gelsolin (GSN) as an important actin-binding protein in promoting HCC invasion and metastasis.

**Methods:**

The relationship between GSN expression and clinical characteristics was observed by immunohistochemistry (IHC). In vitro and in vivo experiments confirmed the role of GSN in HCC metastasis. Dual-immunoprecipitation (IP), immunofluorescence (IF), western blotting, and the gelatinase activity assay were used to investigate the mechanism of GSN-promoting metastasis. PEX fusion proteins were used to intervene in the transfer molecular chain.

**Results:**

Our study found that GSN promoted HCC invasion and metastasis through its synergistic effect with actin-related transfer molecular chain (actin-CD44-MMPs). Concretely, as an important binding molecule of actin, GSN activated MMP2 by interacting with MMP14. Furthermore, CD44 might be a key node in the above-mentioned mechanism. The use of MMP14 domain (PEX fusion protein) to competitively bind to CD44 helped to inhibit the activation of downstream MMP2.

**Conclusions:**

GSN played crucial roles in HCC metastatic process. An improved understanding of the multiple effects of GSN in HCC might facilitate a deeper appreciation of GSN as an important HCC regulator. The study identified GSN and its regulated transfer molecular chain as potential therapeutic targets for HCC.

## Introduction

HCC is one of the most common and aggressive malignancies and the third leading cause of cancer-related deaths [1]. The better understanding of the cellular and molecular mechanisms of HCC will help us to find more advanced treatments. Recently, the influence of cytoskeletal changes in the migration ability of tumor cells is being concerned. GSN, which is a Ca^2+^-dependent actin filament severing and capping protein, has been identified as an important player in tumorigenesis. Abnormal expression of GSN has been observed in many types of cancers [2–5]. The alteration of GSN results in altered actin cytoskeletal architecture and increased cell motility, both of which are essential for tumor cellular migration and invasive growth.

Our results revealed a significant positive correlation between increased GSN and HCC metastatic behavior. It has been reported that GSN could be a biomarker for evaluating the lymph-node metastasis of HCC [6]. Some researchers also considered GSN continuously up-regulated during the entire invasion process [7], promoted HCC progression by regulating epithelial–mesenchymal transition, and correlated with a poor prognosis [8]. Although the above evidence implied a potential role of GSN as a metastasis-associated molecule in HCC, its molecular regulation in the invasion process was still unknown. In this study, we aimed to investigate the mechanism by which GSN acts as an actin-binding protein to promote HCC metastasis.

## Methodology

### Patients and specimens

Tissue samples of HCC and para-carcinoma were obtained from the Affiliated Tumor Hospital of Guangxi Medical University (Nanning, China). The final paraffin-embedded tissue samples were derived from 69 HCC patients.

### Cell culture and recombinant plasmid DNA

The HCC cell line HepG2 was purchased from the Institute of Biochemistry and Cell Biology, Shanghai, China. Expressed or shRNA fractions were connected to pCDH–CMV-MCS-EF1-Puro or pGFP-C-shLenti vectors, respectively. Over-expressed pCDH-GSN, pCDH-MMP14 or inhibited GFP-GSN, GFP-MMP14 plasmids were transfected into HepG2 cells. pCDH-Control and GFP-Control were transfected as contrast.

### IHC

Three consecutive slides of each tissue block were prepared to replicate the experiments. IHC was performed according to the protocol of antibody (Abcam plc, Cambridge, UK). Negative control slides omitting the primary antibodies were included in the assay.

### Wound-healing assay

The experiment was performed by plating 3.5 × 10^5^ cells in 6-well plates and carried out in triplicate. Photographs of cells invading the scratch were taken at the indicated time points. 

### Migration and invasion assay

Cell culture inserts were seeded with 2.5 × 10^4^ cells. The experiments were carried out in triplicate according to the instruction of BD BioCoat^™^ Matrigel^™^ kit (Corning, NY, USA).

### Nude mice model of metastasis

A total of 1 × 10^6^ transfected HepG2 cells were implanted into the caudal vein of 6-week-old male BALB/C allogeneic athymic nude mice. Eight mice were used in each group. The mice were killed and dissected 27 days after tumor inoculation. The metastasis incidence and tumor numbers were counted. Paraffin sections and HE staining were prepared to follow the routine procedures to verify tumorigenesis pathologically.

### Dual-IP

Dual-IP was performed according to the instruction of Flag-HA double tag IP kits (Thermo Fisher Scientific, NY, USA). We extra employed HepG2 cells that were transfected with the pCDH-GSN-FLAG-HA construct. HepG2 cells that expressed pCDH-HA-FLAG without a GSN fragment were served as a control.

### Western blotting

Protein samples were prepared according to the instructions of Mammalian Protein Extraction Kit, Membrane Protein Extraction Kit, and Cytoplasmic Extraction Kit (Thermo Fisher Scientific Co., Ltd, Shanghai, China). Western blotting was performed according to the previous method of our research group [9].

### IF

We extra prepared HepG2 cells that were co-transfected with the pCDH-GSN and pCDH-MMP14 plasmids (GSN/MMP14). A total of 5 × 10^6^ transfected HepG2 cells were planted on the glass slide. Prepared slides were mounted for imaging using a confocal system (Nikon, Tokyo, Japan).

### Non-denaturing electrophoresis of gelatin substrates

The experiment was carried out according to the instructions of Gelatin Zymography Kit (Zhongkeritai Biotechnology Co., Ltd, Beijing, China).

### In-cell western blotting

A total of 5 × 10^6^ transfected cells were inoculated on 96-well plates. The expression of target protein was calculated as the ratio of target protein to GAPDH. The experiment was conducted in three replicates.

### Statistical analysis

SPSS19.0 was used for statistical analysis, and the results were expressed as mean ± SD. Independent sample *t* test was used for comparison between the two groups. Kruskal–Wallis test was used to compare multiple continuous independent samples. Rank sum test was used for pathological change grade data. *P* < 0.05 was considered significant.

## Results

### GSN promotes HCC invasion and metastasis

**Fig. 1:**
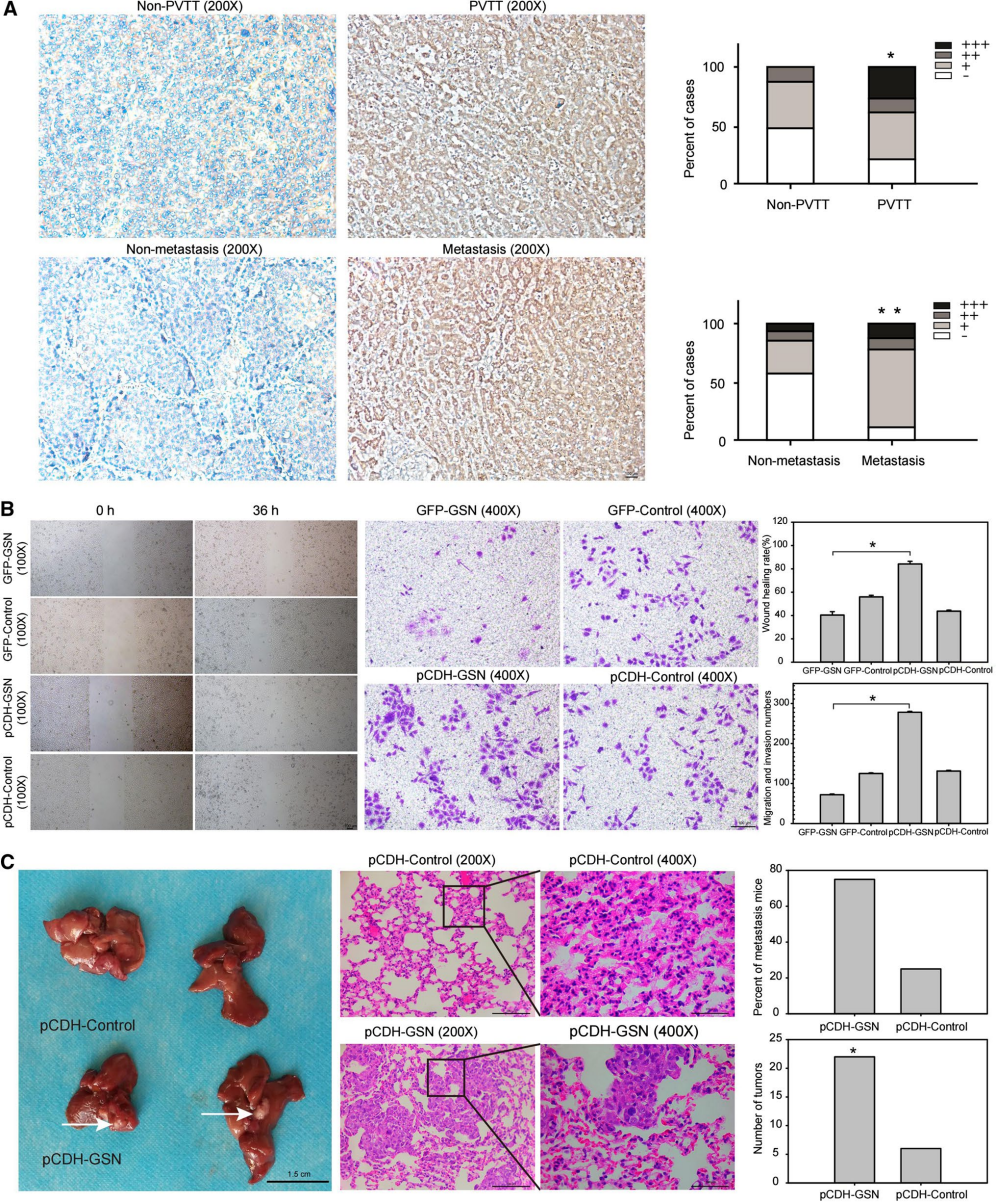
The relationship between GSN expression and HCC metastasis. **A** Expression intensity of positively staining cells: staining cells were divided into different grades (−, + , ++, and +++), *P < 0.05, **P < 0.01. **B** In vitro invasion and metastasis assays, *P < 0.05. **C** Lung gross observation: the white arrows were used to point out the metastatic tumor, *P < 0.05

The clinical characteristics of 69 HCC patients are listed in Table 1. Statistical analysis revealed that GSN expression was higher in patients with PVTT (portal vein tumor thrombus) and metastasis. Patients with HCC metastasis had more positive expression rates (88.9%) than those with non-metastasis (43.2%), **P < 0.01 (Fig. 1A). However, there were no significant differences between GSN expression and the patients’ gender, age, tumor numbers, tumor size, or recurrence. Wound-healing assay showed that GSN promoted cells to move and heal 84.2% of wounded areas while comparing to the shGSN cells with only a 40.4% healing rate, *P < 0.05. In migration and invasion assay, pCDH-GSN group also showed a number of 278 ± 11 migrating cells, which was significantly higher than that of the GFP-GSN (72 ± 5), *P < 0.05 (Fig. 1B). The results of in vivo experiment showed that 75% of the mice which transplanted with pCDH-GSN developed severe lung metastasis. The number of metastasis tumors was also much higher in pCDH-GSN group when compared with the control, *P < 0.05 (Fig. 1C).

**Table 1:** Details of HCC patients used to investigate GSN expression

Test parameter	Cases	GSN				*Z*	*P* value
		−	+	++	+++		
Gender							
Male	66	28	24	7	7	− 1.803	0.1
Female	3	3	0	0	0		
Age (years)							
≤ 60	59	25	22	7	5	− 0.641	0.521
> 60	10	6	2	0	2		
Tumor numbers							
Single	30	15	8	1	6	− 0.785	0.48
Multiple	9	5	4	0	0		
Not recorded	30						
Tumor sizes (mm)							
≤ 10	30	16	7	3	4	− 1.293	0.196
> 10	22	6	11	2	3		
Not recorded	17						
PVTT							
+	10	2	4	1	3	− 2.071	0.038
−	59	29	20	6	4		
Recurrence							
+	25	11	8	1	5	− 0.51	0.61
−	44	20	16	6	2		
Metastasis							
+	18	2	11	2	3	− 2.616	0.009
−	37	21	9	3	4		
Not recorded	17

### GSN interacts with MMP14

**Fig. 2:**
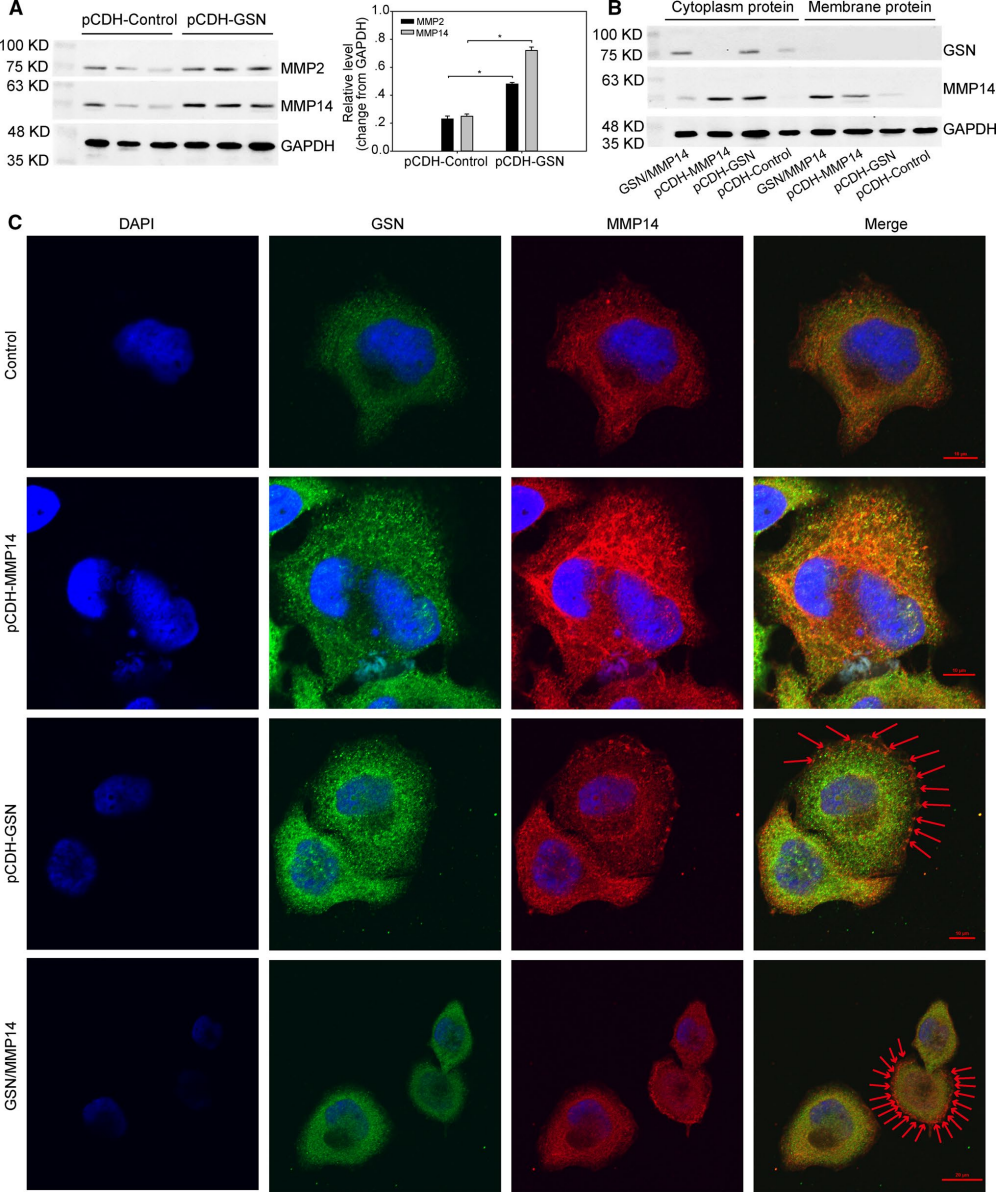
Interaction between GSN and MMP14. **A** The relationship between GSN level and the expression of MMP14 and MMP2, *P < 0.05. **B** The expression and co-expression of GSN, MMP14 in cytoplasm and cell membrane. **C** Subcellular localization of GSN, MMP14 expression and co-expression: the red arrows were used to point out the expression of MMP14 on the cell membrane

Dual-IP suggested that GSN interacted with transfer factor MT-MMP and MMP14 (Table 2). Interestingly, MMP14 belongs to the membrane MMP (MT-MMP) subfamily, and each member of this subfamily contains a potential transmembrane domain, suggesting that MMP14 functions at the cell membrane. It has been reported that MMP14 can activate other members of the MMP family, such as MMP2 [10]. Therefore, we tried to examine if GSN level altered the expression of MMP14 and MMP2. As shown in Fig. 2A, MMP14 and MMP2 were up-regulated in pCDH-GSN cells, *P < 0.05. Then we constructed GSN and MMP14 co-expressed cells (GSN/MMP14) to observe the interaction between GSN and MMP14. We extracted the cytoplasmic proteins and membrane proteins of GSN/MMP14, pCDH-MMP14, pCDH-GSN, and pCDH-Control, respectively, and analyzed the expression of GSN and MMP14 in cytoplasm and membrane. The results showed that GSN was expressed in the cytoplasm rather than cell membrane. When GSN was up-regulated in the cytoplasm, the expression of MMP14 also increased while comparing to the control cells. However, when GSN and MMP14 were co-expressed, the expression of MMP14 was mainly up-regulated in cell membrane (Fig. 2B). Through IF stain (Fig. 2C), we found that MMP14 was not only increased, but also strongly expressed on the cellular surface of the co-expressed cells. The above results suggested that GSN interacted with MMP14 and induced the translocation of MMP14 from cytoplasm to membrane.

**Table 2:** Details of MS/MS identification for CO-IP

Identified protein	Taxonomy	Mass	Matches	Score
hCG1795391	Homo sapiens	6634	17	68
Chain A, solution structure of the bromodomain of peregrin	Homo sapiens	13809	16	66
Membrane-type matrix metalloproteinase	Homo sapiens	66183	18	74
Novel protein	Homo sapiens	74490	10	58
Retron-type reverse transcriptase, RNA-directed DNA polymerase	Homo sapiens	52436	15	55
Matrix metalloproteinase-14	Homo sapiens	66184	25	82

### GSN promotes HCC metastasis through actin-related transfer molecular chain

**Fig. 3:**
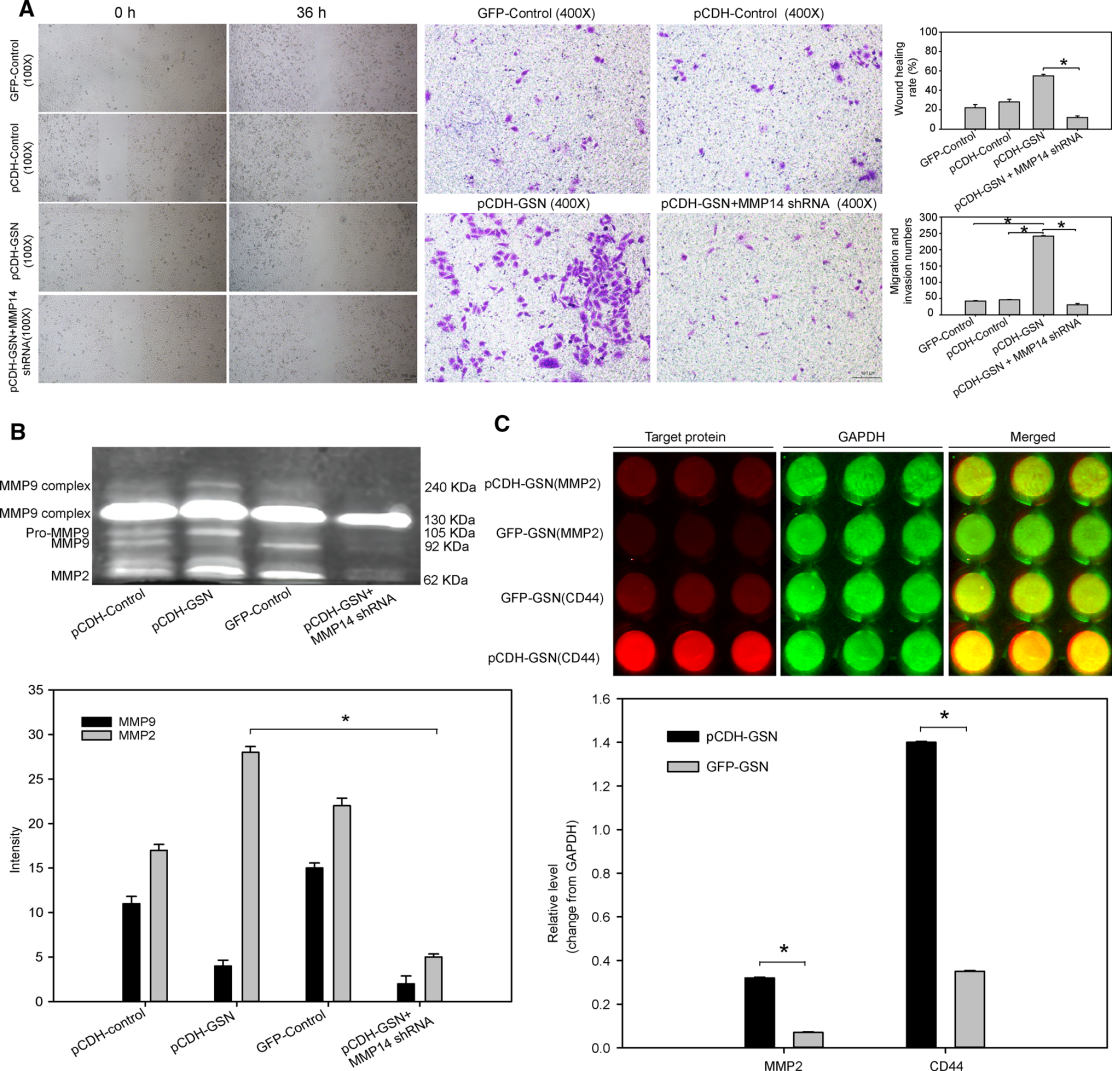
Molecular mechanism of GSN-promoting metastasis. **A** The effect of MMP14 on the ability of GSN to promote metastasis, *P < 0.05. **B** The effect of MMP14 on the activity of MMPs, *P < 0.05. **C** The relationship between CD44, MMP2 level and GSN expression, *P < 0.05, **P < 0.01

We transfected MMP14 shRNA plasmid into pCDH-GSN cells to explore whether MMP14 affects the metastasis function of GSN. Wound-healing assay showed that GSN promoted cells to move and heal 57.4% of wounded areas. However, with MMP14 knocking down, the healing rate was only 10.3%, *P < 0.05. Meanwhile, when MMP14 was down-regulated, the motility and invasiveness of HCC cells were also significantly decreased, *P < 0.05 (Fig. 3A). The possible mechanism pointed to the decreased activity of MMPs (especially MMP2), *P < 0.05 (Fig. 3B).

Previous research reported that the biological process of tumor metastasis depends on the adhesion molecule. Then, we noticed the reports about CD44 (a linker of actin), which involves in actin skeleton remodeling, regulates the cell adhesion and movement, and is directly related to the cell invasion and migration [11, 12]. It has been found that the PEX domain of MMP14 can bind to CD44 on the cell surface, resulting in intracellular cytoskeletal rearrangement as well as cellular migration and invasion, through the activation of MMP2 [13]. Therefore, we tried to explore the relationship between GSN level and the expression of MMP2 and CD44. The results showed that CD44 and MMP2 were up-regulated in GSN over-expressed cells, *P < 0.05, **P < 0.01 (Fig. 3C), which revealed a potential mechanism for GSN promotes HCC metastasis through actin-related transfer molecular chain (atin-CD44-MMPs).

### PEX fusion protein blocks the transfer molecular chain

**Fig. 4:**
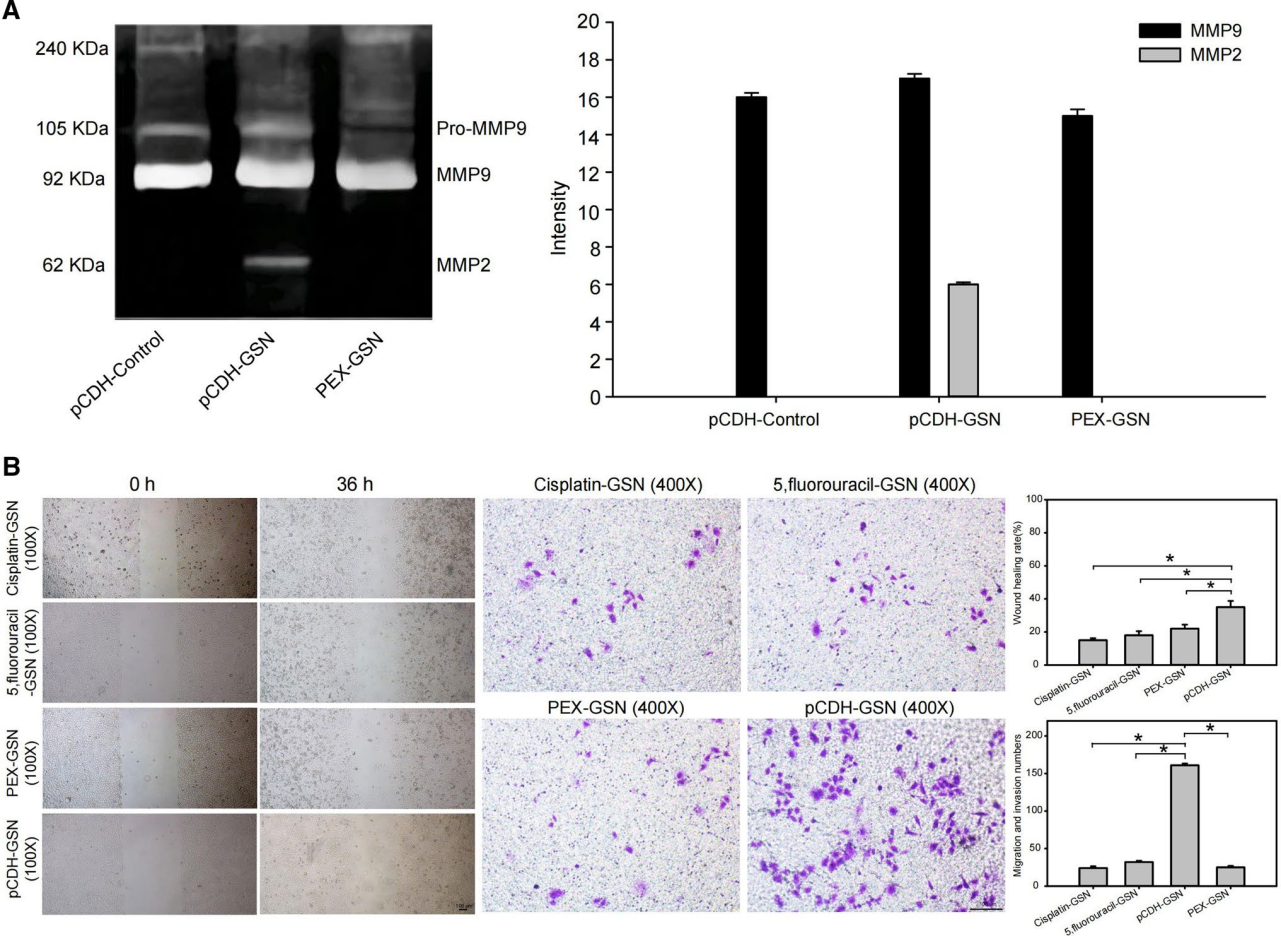
Interventional effect of PEX fusion protein on actin-related transfer molecular chain. **A** PEX fusion protein inhibited the activity of MMP2. **B** PEX fusion protein inhibited the pro-metastatic function of GSN, *P < 0.05

After exploring IC50 by cytotoxicity assay, 30 mM/vehicle PEX fusion protein was acted on pCDH-GSN cells (PEX-GSN). The results showed that PEX inhibited the activity of MMP2 (Fig. 4A). While using cisplatin and 5, fluorouracil as positive controls to analyze the effect of PEX in intervening the transfer molecular chain, the results showed that PEX fusion protein inhibited the metastasis ability of GSN over-expressed cells, *P < 0.05 (Fig. 4B).

## Discussion

GSN is ubiquitously expressed in different cell types and functions as a multifunctional regulator of cell [14]. Here, we focused on its impacts on HCC metastasis. An improved understanding of the function and regulatory mechanisms of GSN may lead to new considerations of this protein as a metastasis regulatory molecule for HCC.

In this study, we revealed the relationship between GSN expression and HCC metastasis. Then, we demonstrated the role of GSN in promoting the invasion and metastasis of HCC and identified a possible mechanism related to transfer molecular chain (actin-CD44-MMPs). Previous studies have found that the movement, migration, adhesion, and invasion behavior of tumor cells are related to actin [15, 16]. Our result suggested that the changes between actin cytoskeleton and extracellular matrix proteins induced to HCC metastasis. This process could be affected by many factors, depended on adhesion molecules, and might be regulated by specific actin-binding factors such as GSN.

We confirmed the interaction between GSN and MMP14. Increased expression of MMP14 in tumor cells enhances the growth, invasion, and metastasis of tumor [17]. MMP14 is generally considered pro-invasive and pro-tumorigenic, because: (1) the expression and activity of MMP14 is elevated in tumor cells and (2) high levels of MMP14 directly correlate with enhanced cell migration. (3) MMP14 can activate other MMP family members [18, 19]. In this paper, we speculated that MMP14 and MMP2 might be the terminal molecules in GSN-mediated transfer mechanism.

CD44, a member of the adhesion molecule family, has been proved to be a linker of actin [20]. Literature has shown that CD44 is related to the tumor metastasis caused by changes in actin cytoskeleton [21, 22]. Furthermore, CD44 could specifically interact with the PEX sequence of MMP14, activate MMP2, and regulate cell migration, suggesting that CD44 and MMP2 may be closely related to the tumor metastasis. Here, we considered that CD44 might be a key node in actin-related transfer molecular chain and act together with actin and MMP family members on the biological processes of HCC metastasis.

## Conclusion

In conclusion, we found that GSN played crucial roles in HCC metastatic processes. Earlier studies of GSN that focused on HCC metastasis are now being extended to mechanisms by which GSN is involved in metastatic behavior. This study identified GSN and its regulated actin transfer molecular chain as potential therapeutic targets for HCC.

## Abbreviations

HCC: Hepatocellular carcinoma
GSN: Gelsolin
IHC: Immunohistochemistry
IP: Immunoprecipitation
IF: Immunofluorescence
MMP: Matrix metalloproteinase
ICC: Immunocytochemistry
PVTT: Portal vein tumor thrombus
MT-MMP: Membrane-type 1 matrix metalloproteinase
